# Polygenic risk score across distinct colorectal cancer screening outcomes: from premalignant polyps to colorectal cancer

**DOI:** 10.1186/s12916-021-02134-x

**Published:** 2021-11-08

**Authors:** Mireia Obón-Santacana, Anna Díez-Villanueva, Maria Henar Alonso, Gemma Ibáñez-Sanz, Elisabet Guinó, Ana López, Lorena Rodríguez-Alonso, Alfredo Mata, Ana García-Rodríguez, Andrés García Palomo, Antonio J. Molina, Montse Garcia, Gemma Binefa, Vicente Martín, Victor Moreno

**Affiliations:** 1 Unit of Biomarkers and Suceptibility (UBS), Oncology Data Analytics Program (ODAP), Catalan Institute of Oncology (ICO), L’Hospitalet del Llobregat, 08908 Barcelona, Spain; 2 ONCOBELL Program, Bellvitge Biomedical Research Institute (IDIBELL), L’Hospitalet de Llobregat, 08908 Barcelona, Spain; 3 Consortium for Biomedical Research in Epidemiology and Public Health (CIBERESP), 28029 Madrid, Spain; 4 Gastroenterology Department, Bellvitge University Hospital, L’Hospitalet de Llobregat, Spain; 5 Digestive System Service, Moisés Broggi Hospital, Sant Joan Despí, Spain; 6 Endoscopy Unit, Digestive System Service, Viladecans Hospital-IDIBELL, Viladecans, Spain; 7 Servicio de Oncología, Complejo Asistencial Universitario de León, 24071 León, Spain; 8 The Research Group in Gene - Environment and Health Interactions (GIIGAS)/Institut of Biomedicine (IBIOMED), Universidad de León, 24071 León, Spain; 9 Faculty of Health Sciences, Department of Biomedical Sciences, Area of Preventive Medicine and Public Health, Universidad de León, 24071 León, Spain; 10 Cancer Screening Unit, Cancer Prevention and Control Program, Catalan Institute of Oncology, L’Hospitalet de Llobregat, Barcelona, Spain; 11 Early Detection of Cancer Research Group, EPIBELL Program, Institut d’Investigació Biomèdica de Bellvitge (IDIBELL), L’Hospitalet de Llobregat, Barcelona, Spain; 12 Department of Clinical Sciences, Faculty of Medicine, University of Barcelona, 08007 Barcelona, Spain

**Keywords:** Polygenic risk score, Colorectal cancer, Screening, Positive fecal immunochemical test, Positive predictive value, Negative predictive value

## Abstract

**Background:**

Different risk-based colorectal cancer (CRC) screening strategies, such as the use of polygenic risk scores (PRS), have been evaluated to improve effectiveness of these programs. However, few studies have previously assessed its usefulness in a fecal immunochemical test (FIT)-based screening study.

**Methods:**

A PRS of 133 single nucleotide polymorphisms was assessed for 3619 participants: population controls, screening controls, low-risk lesions (LRL), intermediate-risk (IRL), high-risk (HRL), CRC screening program cases, and clinically diagnosed CRC cases. The PRS was compared between the subset of cases (*n* = 648; IRL+HRL+CRC) and controls (*n* = 956; controls+LRL) recruited within a FIT-based screening program. Positive predictive values (PPV), negative predictive values (NPV), and the area under the receiver operating characteristic curve (aROC) were estimated using cross-validation.

**Results:**

The overall PRS range was 110–156. PRS values increased along the CRC tumorigenesis pathway (Mann-Kendall *P* value 0.007). Within the screening subset, the PRS ranged 110-151 and was associated with higher risk-lesions and CRC risk (OR_D10vsD1_ 1.92, 95% CI 1.22–3.03). The cross-validated aROC of the PRS for cases and controls was 0.56 (95% CI 0.53–0.59). Discrimination was equal when restricted to positive FIT (aROC 0.56), but lower among negative FIT (aROC 0.55). The overall PPV among positive FIT was 0.48. PPV were dependent on the number of risk alleles for positive FIT (PPVp10-p90 0.48–0.57).

**Conclusions:**

PRS plays an important role along the CRC tumorigenesis pathway; however, in practice, its utility to stratify the general population or as a second test after a FIT positive result is still doubtful. Currently, PRS is not able to safely stratify the general population since the improvement on PPV values is scarce.

**Supplementary Information:**

The online version contains supplementary material available at 10.1186/s12916-021-02134-x.

## Background

Colorectal cancer (CRC) is the third most common cancer type diagnosed in the world and the second deadly cancer globally [1]. Screening strategies reduce the incidence and mortality of CRC by detecting premalignant polyps and cancers at an earlier stage. Compared to no CRC screening, all screening modalities (i.e., fecal occult blood test (FOBT), endoscopy screening, computed tomographic colonography, stool DNA test) provide additional years of life at a cost that is deemed acceptable by most industrialized nations [2]. However, the FOBT that checks stool samples for small amounts of blood has been adopted globally [3]. Among FOBT, the fecal immunochemical test (FIT) is often preferred as it does not require dietary restrictions before sample collection and has higher sensitivity and a lower incidence of interval CRC after a negative screening result [4]. In Spain, CRC screening started in 2000 in Catalonia, and it extended nationally gradually. Currently, the target population for CRC screening is aged between 50 to 69 years, uses the FIT, and reached participation rates of 45.3% in 2016 with a slow increasing trend [5].

Over the past decade, and thanks to extensive genome-wide association studies (GWASs), researchers have identified a significant number of loci associated with CRC risk [6, 7]. In CRC, different approaches to generate predictive polygenic risk scores (PRS) from GWASs have been developed and have offered a way for risk-stratified CRC screening and other targeted interventions [8]. Based on more than 120,000 European ancestry subjects, the largest and most recent GWAS study in CRC built a polygenic risk score that incorporated 140 single nucleotide polymorphisms (SNPs). The area under the receiver operating characteristics curve (aROC) was 0.63 on the discovery population [6]. Thus far, most studies have essentially included clinically diagnosed CRC cases. However, in order to tailor effective CRC early detection and preventive interventions, PRS should also include premalignant polyps, ideally identified in CRC screening population. To the best of our knowledge, only three studies have previously evaluated the usefulness of PRS in a CRC colonoscopy-based screening context, which used PRS of 39, 82, and 22 SNPs, respectively [9–11].

In this cross-sectional study, we aimed to elucidate, in a sample of participants in a FIT-based screening program, the value of a PRS using the most recent CRC loci (*n* = 133) to stratify individuals according to their risk of developing distinct colorectal cancer screening outcomes, from premalignant polyps to colorectal cancer. A second aim of the study was to elucidate the utility of the PRS as a second test after a FIT positive result to indicate or prioritize a colonoscopy.

## Methods

### Study population and sample collection

This study population consisted of 3619 participants from 2 observational studies for whom genetic data were available: Colorectal Cancer Genetics & Genomics (CRCGEN; *n* = 1801) and Colorectal Cancer Screening (COLSCREEN; *n* = 1818). CRCGEN combines data of two Spanish case-control studies. The first one, conducted in the University Hospital of Bellvitge, L’Hospitalet del Llobregat, (Barcelona), recruited incident pathology-confirmed CRC cases (*n* = 304) and age and sex frequency-matched hospital controls (*n* = 293) during the period 1996–1998. The control group was randomly selected among patients without previous CRC admitted to the same hospital during the same period. To avoid selection bias, the criterion of inclusion in the control group was a new diagnosis. The second study was conducted in parallel in the University Hospital of Bellvitge and in the Hospital of León, León, during 2007–2015 and recruited a total of 633 incident CRC cases (313 Bellvitge and 320 León) and 571 population controls (free of CRC, 164 Bellvitge and 407 León). The control group was recruited by inviting to participate subjects selected from the primary health care lists of the hospitals’ referral areas, frequency matched by age and sex. COLSCREEN is a cross-sectional screening cohort study designed to recruit participants from the ongoing population-based CRC screening program conducted by the Catalan Institute of Oncology, L’Hospitalet del Llobregat (Barcelona), from 2011 to 2020. The design of the CRC screening program, based on FOBT, has been published elsewhere [12, 13]. Exclusion criteria to participate at the biennial screening program were as follows: gastrointestinal symptoms; family history of hereditary or familial colorectal cancer, personal history of CRC, adenomas or inflammatory bowel disease; and colonoscopy in the previous 5 years or a FIT within the last 2 years, terminal disease, and severe disabling conditions. Most of the participants of the COLSCREEN study were invited to participate after a positive FIT result (*n* = 1242, 77%; ≥ 20 μg Hb/g feces), but we also invited to undergo a colonoscopy to a sample of 362 subjects with a negative FIT result (< 20 μg Hb/g feces). To increase CRC sample size, we further included in the COLSCREEN study 70 newly diagnosed CRC identified by the hospital CRC Functional Unit. These clinically diagnosed CRC cases, though labeled under COLSCREEN because they were recruited simultaneously to that study, for analyses were combined with others of CRCGEN and were excluded when the analyses were restricted to participants in screening.

Colonoscopy and/or CRC histological reports were examined and used to classify COLSCREEN participants into different categories following the proposal by Castells et al. for risk stratification of patients with CRC and/or serrated polyps [14]: low-risk lesions (LRL; *n* = 286), intermediate-risk lesions (IRL; *n* = 352), high-risk lesions (HRL; *n* = 226), and CRC screening program cases (*n* = 70). Controls (free of CRC) were classified into population-based controls and screening controls (normal colonoscopy or no-risk lesions).

All participants who agreed to take part of the CRCGEN and COLSCREEN studies provided written informed consent and donated a blood sample at recruitment. Each hospital’s ethics committees (University Hospital of Bellvitge and Hospital of León) approved the protocols of the studies (PR148/08, PR073/11, PR084/16).

### Genotyping and single nucleotide polymorphism data

Genotyping within the CRCGEN study was conducted in 2016 from blood DNA using the Infinium OncoArray-500 K BeadChip (Illumina, San Diego, CA) which contains 500,000 SNPs, whereas the genotyping within the COLSCREEN study was performed in 2019 using the Infinium Global Screening Array v2.0 (Illumina, San Diego, CA) which includes near 800,000 SNPs markers. The correlation of the minor allele frequency (MAF) between arrays was excellent (Pearson *r* > 0.99).

SNPs were filtered out if Hardy Weinberg equilibrium *p* value was < 1e−04 or if the MAF was < 0.001. Multiallele SNPs and SNPs outside autosomes or chromosome X or with > 5% of missing values were also excluded. Likewise, duplicated, or related samples and samples with > 1% of missing values or with no sex concordance were also excluded. Whole genome imputation was performed using Michigan Imputation Server [15], for each dataset separately, using the Haplotype Reference Consortium panel (HRC.r1.1.2016) for CEU population as reference.

In 2020, Thomas et al. reported 140 SNPs associated to CRC risk in a large-scale GWAS study [6]. For the present study, a total of 133 SNPs were used to calculate the PRS. One SNP was not found in our data after imputation (rs6928864) and six SNPs (rs35470271, rs145364999, rs755229494, rs77969132, rs373585858, and rs556532366) were excluded due to low imputation quality information index (*R*^2^ < 0.3). None of the 133 SNPs were in linkage disequilibrium (Additional file 1: Tab. S1).

### Statistical analysis

Odds ratios (OR) and 95% confidence intervals (95% CI) were estimated using unconditional logistic regression models to evaluate associations between each analyzed variant and the outcome, defined as cases (IRL, HRL or CRC, either screening or clinical) versus controls (normal colonoscopy, LRL or population control). All samples were combined for this analysis and, previously, potential confounders were explored (age, sex, genetic ancestry, family history and array). Though the crude and fully adjusted models provided very similar OR estimates, we report the adjusted ORs.

A principal component analysis (PCA) with the ancestry-informative marker SNPs (AIMS) including 1397 HapMap samples was performed [16]. Based on ethnicity of HapMap samples, we could classify our samples by ancestry: European (*n* = 3509), Latino (*n* = 90), and African (*n* = 20) (Additional file 2: Fig. S1). We decided not restrict the sample to European ancestry, since the 110 non-European samples had a minimal impact in the estimates and this population also participates in CRC screening. We adjusted the analyses by the first 5 PCs, though only the first two were associated with the outcome. We also performed a sensitivity analysis excluding the subjects with no European ancestry.

To assess genetic susceptibility, two approaches were used: an unweighted PRS and a weighted PRS (w-PRS). Each SNP was coded as 0, 1, or 2 copies of the risk allele. The PRS was defined as the sum of risk alleles across all 133 SNPs. The w-PRS was assessed using the published *β* values reported by Thomas et al. as weights. However, because part of our data (CRCGEN) was used by Huyghe et al. as a discovery data [7], the unweighted PRS was preferred, though the weights used had been corrected for the winner’s course bias.

The PRS was analyzed initially as response in a multivariate linear model to assess potential confounding. Though only sex and two ancestry components were significant, we calculated and adjusted PRS as the residuals of the linear model that included sex, age, five ancestry components, array, and family history of CRC. Then, we used that adjusted PRS to estimate averages for the different risk groups (population control, screening control, LRL, IRL, HRL, screening CRC, and clinically diagnosed CRC). Differences between PRS mean values within variables (ethnicity, sex, age, and FIT) were assessed using Student’s *t* test. To identify distributional changes across groups, the non-parametric Mann-Kendall Trend test was used [17, 18]. Stratified analysis by sex and age (< 60, ≥ 60) were also carried out.

Further, we performed additional analyses focusing only on screening samples, which were dichotomized into cases with pathogenic lesions (*n* = 648; IRL, HRL, and screening CRC) and controls (*n* = 956; LRL and screening controls). Additional stratified analyses by sex, age (< 60, ≥ 60), and FIT (positive FIT, negative FIT) were conducted within this subset. We carried out two exploratory analyses in order to verify the consistency of the results. First, we used the w-PRS instead of the PRS, and second, cases were defined as HRL and screening CRC (*n* = 296) and controls included the IRL group, with LRA and screening controls (*n* = 1308).

The predictive accuracy of the models to discriminate cases and controls was assessed with sensitivity, specificity and, aROC as implemented in the pROC R package [19]. To reduce the impact of estimating the model coefficient in our data, we used fivefold cross-validation to calculate the aROC. The *roc.test* function with DeLong test from the same package was used to compare two aROC curves. Utility of the PRS was assessed calculating the positive predictive values (PPV) and negative predictive values (NPV). Since our sample was not representative of the prevalence of lesions in average risk population, because most of the subjects had been selected by a previous FIT test, an estimate of the population PPV and NPV were calculated using a weighted average of the values calculated by strata according to FIT result. Based on the number of participants with a positive FIT result in the population screening program, sampling weights of 0.06 and 0.94 were applied for participants with a positive and a negative FIT result, respectively [13].

Lastly, a quantile plot was performed stratifying the screening population according to the adjusted PRS. Participants were categorized in deciles based on the distribution in the control group. The first PRS decile was treated as reference category. The OR and the corresponding 95% CI for the association between PRS and CRC (including IRL, HRL, and CRC) risk were estimated using unconditional logistic regression models.

All statistical analyses and graphical representations were carried out using R statistical software (R Foundation for Statistical Computing, Vienna, Austria) and SNP selection and PRS calculations were performed using PLINK version 1.9 [20]. All statistical tests were two-sided and statistical significance was set at *α* = 5%.

## Results

### Characteristics of the study population and scoring by PRS

Main characteristics of the study population are presented in Table 1. Participants were classified into 7 groups according to their status: 1008 population controls, 670 screening controls, 286 LRL, 352 IRL, 226 HRL, 70 screening CRC, and 1007 clinically diagnosed CRC (937 from CRCGEN and 70 from COLSCREEN). The mean age was 63.1 years and 43% were female.

**Table 1:** Characteristics of the overall study population stratified by categories following the classification by Castells et al.

		Study		Sex	Family history	Age (year)			PRS			Weighted PRS		
	***n*** (%)	CRCGEN ***n*** (%)	COLSCREEN ***n*** (%)	Female ***n*** (%)	Yes^a^ ***n*** (%)	Min	Mean (SD)	Max	Min	Mean (SD)	Max	Min	Mean (SD)	Max
**Population control**	1008 (28)	864 (48)	144 (8)	467 (30)	63 (20)	19	64.2 (12.0)	92	111	131.7 (7.2)	156	5.7	7.3 (0.4)	8.9
**Screening control**	670 (19)	–	670 (37)	406 (26)	48 (15)	49	59.5 (6.0)	70	111	131.5 (7.1)	149	6.0	7.2 (0.4)	8.7
**LRL**	286 (8)	–	286 (16)	125 (8)	24 (8)	49	60.3 (5.7)	71	110	131.8 (7.4)	151	6.0	7.3 (0.5)	8.4
**IRL**	352 (10)	–	352 (19)	126 ()8	20 (6)	49	59.8 (5.4)	70	111	132.6 (7.0)	151	6.0	7.4 (0.5)	8.7
**HRL**	226 (6)	–	226 (12)	65 (4)	15 (5)	50	60.6 (5.9)	70	114	133.7 (7.4)	150	6.2	7.4 (0.5)	8.5
**Screening CRC**	70 (2)	–	70 (4)	22 (1)	–	50	61.1 (5.9)	69	119	133.9 (6.4)	150	6.5	7.3 (0.4)	8.3
**Clinically diagnosed CRC**	1007 (28)	937 (52)	70 (4)	353 (23)	146 (46)	23	67.3 (11.0)	91	110	134.5 (7.0)	156	6.1	7.4 (0.4)	8.9
**Total**	3619	1801 (50)	1818 (50)	1564 (43)	316 (9)	19	63.1 (9.9)	92	110	132.7 (7.2)	156	5.7	7.3 (0.5)	8.9

*CRC* colorectal cancer; *CRCGEN* Colorectal Cancer Genetics & Genomics study; *COLSCREEN* The Colorectal Cancer Screening Study; *HRL* high-risk lesion; *IRL* intermediate risk lesion; *LRL* low risk lesion; *PRS* adjusted polygenic risk score.

^a^Percentages may differ due to missing values (*n* = 84)

The association between CRC and the analyzed variants is presented in Additional file 1: Tab. S1. The analysis included all the samples and was adjusted by sex, age, array, family history, and centered PC1-PC5. In this analysis, controls were population and screening controls and, LRL and cases were IRL, HRL, screening CRC, and clinically diagnosed CRC. Among the 133 included SNPs, only 24 were statistically significant (*P* value< 0.05) associated with the outcome.

**Fig. 1:**
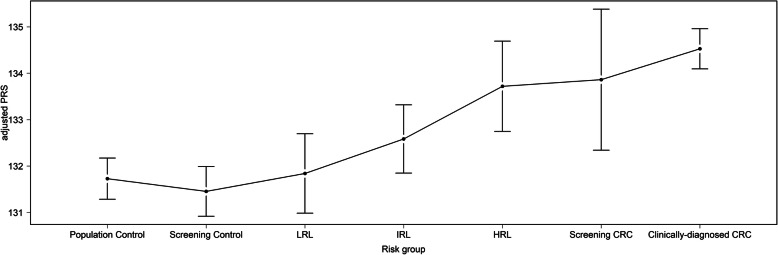
Distribution of the adjusted polygenic risk score according to the different 7 risk groups. Data are represented as the mean and 95% CI for each group

The mean (SD) value for the PRS was 132.7 (7.2). Figure 1 shows the distribution of the PRS across the different 7 groups. The mean PRS values increased along the CRC tumorigenesis pathway (Mann-Kendall *P* value 0.007): from 131.7 to 134.5 for population controls and clinically diagnosed CRC, respectively. No statistical differences in PRS values were observed by sex and by age (< 60, ≥ 60) either globally or across the 7 groups (Additional file 2: Fig. S2 and Fig. S3).

The analysis using a w-PRS yelled to similar distributions across groups (Mann-Kendall *P* value 0.07) compared to the PRS, except for the screening CRC group (Additional file 2: Fig. S4). Likewise, a sensitivity analysis evaluating ethnicity was conducted. Non-European ancestry samples accounted for 3% of the total participants (*n* = 110) and were unequally distributed between the different 7 groups: 13 population controls, 54 screening controls, 16 LRL, 16 IRL, 2 HRL, 1 screening CRC, and 7 clinically diagnosed CRC. Mean PRS values were not statistically significant different between European and non-European participants (132.7 vs. 133.0; *t* test *P* value 0.66). The PRS distribution across the 7 groups remained unchanged when non-European samples were excluded from the analysis (Mann-Kendall *P* value 0.007) (Additional file 2: Fig. S5).

### Risk stratification for screening population according to PRS

**Fig. 2:**
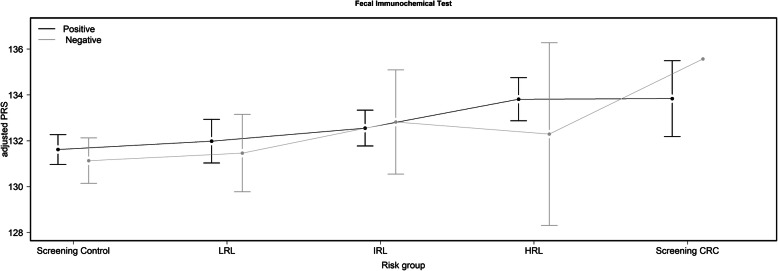
Distribution of the adjusted polygenic risk score according to the different 5 groups by fecal immunochemical test result. Only screening samples are represented in this figure. Data are represented as the mean and 95% CI for each risk group. Negative fecal immunochemical test defined as < 20 μg Hb/g feces. Positive fecal immunochemical test defined as ≥ 20 μg Hb/g feces

The analyses performed to the subset of screening population included a total of 1604 participants. Descriptive characteristics of this subset are presented in Table 2. The mean age was 59.9; 46% were female and 77% had a positive FIT result. Only one CRC was detected among the 362 subjects with negative FIT result. The PRS including only screening population ranged from 110 to 151, with a mean (SD) value of 132.2 (7.2). Regarding differences in PRS values by FIT, participants with a negative FIT result had statistically significant lower mean PRS values than positive FIT participants (132.4 in negative FIT vs. 131.5 in positive FIT; *t* test *P* value 0.03); however, no differences were observed comparing FIT means across screening groups (all Bonferroni-adjusted *t* test *P* values = 1) (Fig. 2). Stratified analysis by age (< 60 vs. ≥ 60) showed similar PRS mean values (132.1 vs. 132.3; respectively), and no statistically significance difference was observed neither overall (*t* test *P* value 0.68) nor across the 5 screening groups (data not shown).

**Table 2:** Characteristics of the CRC screening participants stratified by categories following the classification by Castells et al.

		Study		Sex	Family history	Age (year)			FIT		PRS			Weighted PRS		
	***n*** (%)	CRCGEN ***n*** (%)	COLSCREEN ***n*** (%)	Yes ***n*** (%)	Yes^a^ ***n*** (%)	Min	Mean (SD)	Max	Negative FIT^b^ ***n*** (%)	Positive FIT^c^ ***n*** (%)	Min	Mean (SD)	Max	Min	Mean (SD)	Max
**Screening control**	670 (42)	–	670 (42)	406 (25)	48 (45)	49	59.5 (6.0)	70	226 (62)	444 (36)	111	131.5 (7.1)	149	6.0	7.2 (0.4)	8.7
**LRL**	286 (18)	–	286 (18)	125 (8)	24 (22)	49	60.3 (5.7)	71	78 (22)	208 (17)	110	131.8 (7.4)	151	6.0	7.3 (0.5)	8.4
**IRL**	352 (22	–	352 (22	126 (8)	20 (19)	49	59.8 (5.4)	70	43 (12)	309 (25)	111	132.6 (7.0)	151	6.0	7.4 (0.5)	8.7
**HRL**	226 (14)	–	226 (14)	65 (4)	15 (14)	50	60.6 (5.9)	70	14 (3.9)	212 (17)	114	133.7 (7.4)	150	6.2	7.4 (0.5)	8.5
**Screening CRC**	70 (4)	–	70 (4)	22 (1)	–	50	61.1 (5.9)	69	1 (0.1)	69 (6)	119	133.9 (6.4)	150	6.5	7.3 (0.4)	8.3
**Total**	1604	–	1604	744 (46)	107 (7)	49	59.9 (5.8)	71	362 (23)	1242 (77)	110	132.2 (7.2)	151	6.0	7.3 (0.5)	8.7

*CRC* colorectal cancer; *CRCGEN* Colorectal Cancer Genetics & Genomics study; *COLSCREEN* The Colorectal Cancer Screening Study; *FIT* fecal immunochemical test; *HRL* high-risk lesion; *IRL* intermediate risk lesion; *LRL* low risk lesion; *PRS* adjusted polygenic risk score

^a^Percentages may differ due to missing values (*n* = 38)

^b^Negative FIT defined as < 20 μg Hb/g feces

^c^Positive FIT defined as ≥ 20 μg Hb/g feces

To assess the discriminative accuracy of the PRS, measured by the aROC, the screening population were categorized into cases, including diagnosis of IRL, HRL, or screening CRC (*n* = 648), and controls, including LRL and screening controls (*n* = 956). Additional file 4: Tab. S2 shows the sample size for the distribution of the PRS in cases and controls for each model. Further, detailed PPV and NPV and their correspondent 95%CI are listed in Additional file 4: Tab. S3.

**Fig. 3:**
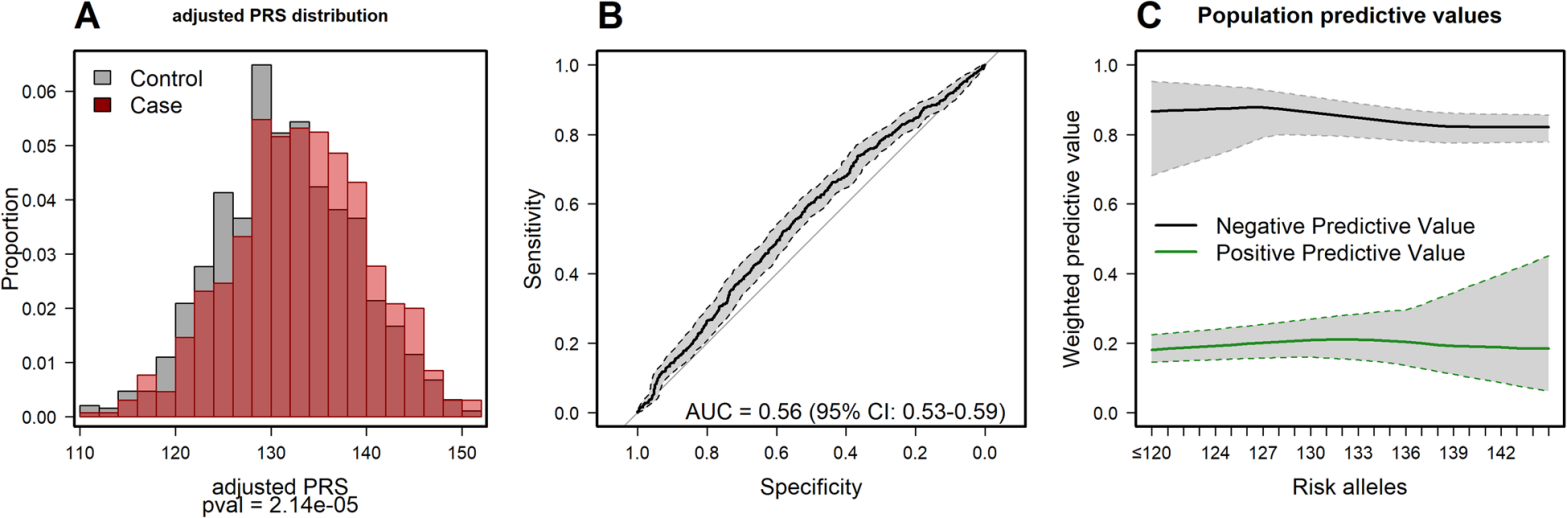
**A** Distribution of the adjusted polygenic risk score in screening cases and controls. **B** Receiver operating characteristic curve based on 133 SNPs used to measure the area under the curve in cases compared to controls. **C** Positive and negative predicted values for the number of CRC risk alleles weighted by fecal immunochemical test (positive weight = 0.06 and negative weight = 0.94)

Figure 3A shows the distribution of PRS in screening cases and controls, which approximated normal distributions. The mean PRS was 133.1 for cases and 131.6 for controls. The cross-validated aROC was 0.56 (95% CI 0.53–0.59) (Fig. 3B). The PPV at 10th PRS percentile was 0.19 (95% CI 0.15–0.24) but slightly increased at the 90th percentile, though the 95%CI was very wide (PPV_p90_ 0.21, 95% CI 0.10–0.39). Otherwise, the NPV decreased with increasing number of risk alleles (NPV_p10-p90_ 0.88–0.82) (Fig. 3C).

The predictive accuracy was 0.57 (95% CI 0.55–0.60) when the w-PRS for cases and controls was evaluated (Additional file 2: Fig. S6). Results from the analysis performed in high-risk cases restricted to HRL and CRC showed a predictive accuracy of the PRS of 0.58 (95% CI 0.54–0.61); however, this increment was not statistically significant compared to the main model (DeLong test *P* value 0.54). The NPV at the 10th percentile was 0.97 (95% CI 0.87–0.99) (Additional file 2: Fig. S7).

Increased risks for cases (IRL, HRL, or screening CRC) were observed starting from the seventh decile (OR_D7vsD1_ 1.68, 95% CI 1.06–2.65). Participants classified at the highest decile were at 1.9-fold risk compared to the lowest decile (OR_D10vsD1_ 1.92, 95% CI 1.22–3.03; Mann-Kendall *P* value: 0.002) (Additional file 2: Fig. S8).

The corresponding curves of predictive values according to the number of risk alleles by sex are shown in Additional file 2: Fig. S9. The aROC was 0.57 (95% CI 0.53–0.62) for women and 0.56 (95% CI 0.52–0.59) for men, with overlapping confidence intervals (DeLong test *P* value: 0.59) (Additional file 2: Fig. S9 B S9 E, respectively). Despite that in both scenarios NPV decreased with increasing number of risk alleles, lower NPV were observed in men (NPV_p10-p90_ 0.83–0.76) than in women (NPV_p10-p90_ 0.92–0.88). PPV increased with increasing number of risk alleles among women (PPV_p10-p90_ 0.14–0.21), but not in men (PPV_p10-p90_ 0.25–0.21) (Additional file 2: Fig. S9 C S9 F).

Two age groups among screening participants were defined as < 60 years (286 cases and 472 controls) and ≥ 60 years (362 cases and 484 controls). For elderly participants, the PRS improved the predictive accuracy by 0.01 points (aROC 0.57; 95% CI 0.54–0.61), whereas no improvement could be observed for younger participants (aROC 0.54; 95% CI 0.50–0.59) (Additional file 2: Fig. S10 E and S10 B, respectively). For elderly participants, the 90th PRS percentile PPV was 0.47 and NPV was 0.81, whereas for younger participants the 90th PRS percentile PPV was 0.16 and NPV was 0.93 (Additional file 2: Fig. S10 F and S10 C, respectively).

**Fig. 4:**
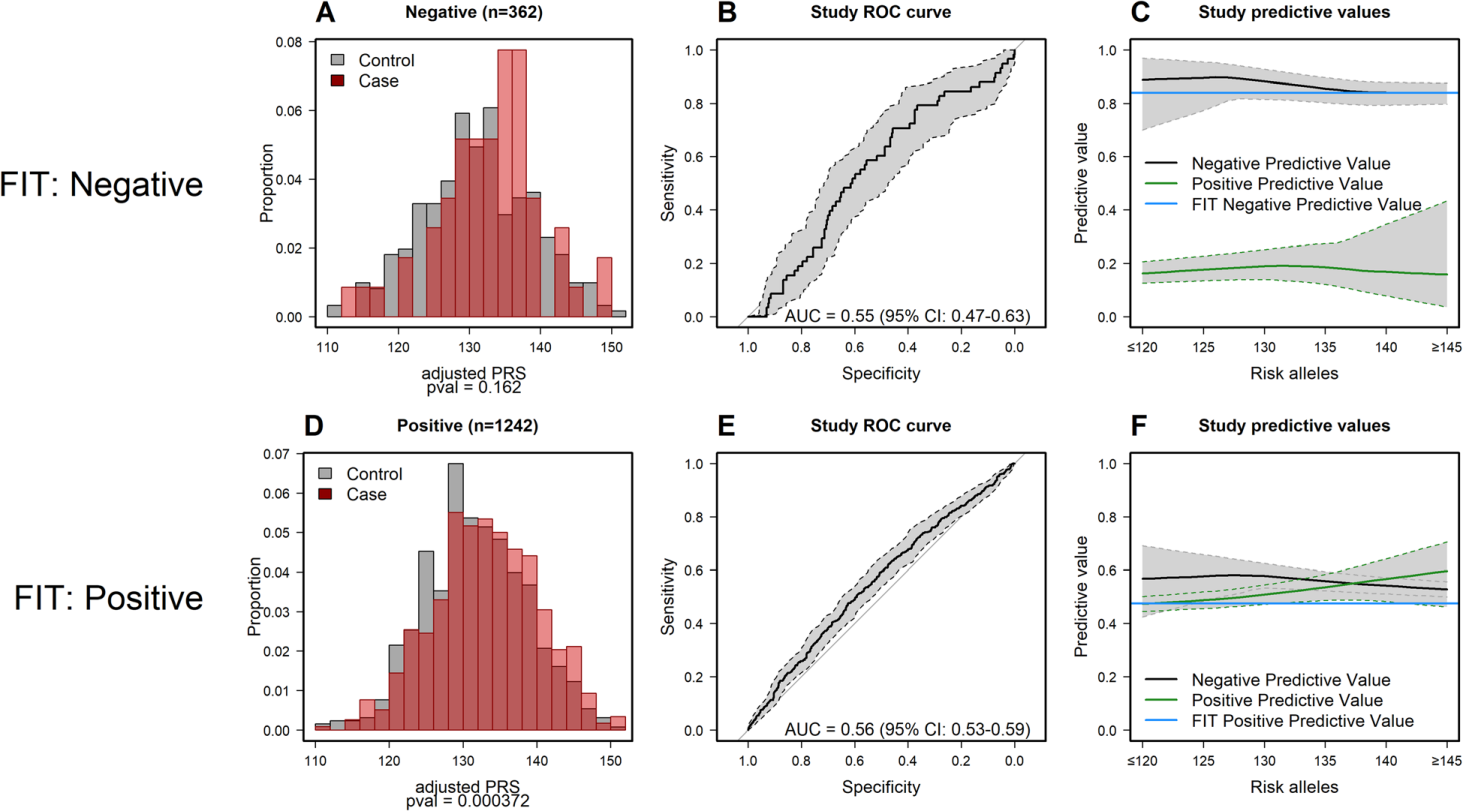
**A** Distribution of the adjusted polygenic risk score among negative fecal immunochemical test (FIT) result in cases and controls. **B** Receiver operating characteristic curve for based on 133 SNPs used to measure the area under the curve in negative FIT cases compared to negative FIT controls. **C** Positive and negative predicted values for the number of CRC risk alleles in negative FIT. The overall negative predictive value is 0.84. **D** Distribution of the polygenic risk score among positive FIT in cases and controls. **E** Receiver operating characteristic curve based on 133 SNPs used to measure the area under the curve in positive FIT cases compared to positive FIT controls **F** Positive and negative predicted values for the number of CRC risk alleles in positive FIT. The overall positive predictive value is 0.47

PRS was also assessed separately for positive FIT (590 cases and 652 controls) and negative FIT (58 cases and 304 controls) (Fig. 4). Discrimination was equal when restricted to positive FIT (aROC 0.56, 95% CI 0.53–0.59) and lower among negative FIT with an aROC of 0.55 (95% CI 0.47–0.63) (Fig. 4E and B, respectively). The NPV and PPV were dependent on the number of risk alleles for positive FIT (NPV_p10-p90_ 0.56–0.54; PPV_p10-p90_ 0.48–0.57). PPV also increased with increasing number of risk alleles for negative FIT (PPV_p10-p90_ 0.17–0.19) (Fig. 4C and F, respectively).

## Discussion

In the present study, we assessed a PRS based on 133 of the 140 independent GWAS SNPs previously reported in association to CRC [6]. An increasing PRS trend was observed along the stepwise progression from healthy individuals, LRL, IRL, and HRL to cancer of the colorectum on a sample of 3619 participants.

Our study included a subset of 1604 participants recruited from a population-based CRC Screening Program. We found that the PRS was clearly associated with increased CRC risk (including diagnosis of IRL, HRL, or CRC), although the predictive accuracy of the PRS was low (aROC 0.56). The aROC improved by 0.02 when the model was restricted to HRL and CRC; however, this increment was not significant, the sample size was modest, and the confidence intervals were wide. In this sense, the prediction from the present study falls within the range reported by previously published articles, with aROC between 0.56 and 0.63 [6, 9, 10, 21–25]. However, a study conducted within The Korean Cancer Prevention Study-II reported the highest aROC values so far, 0.69, for a model including age and the PRS among men. It is important to take into consideration that the SNPs that were selected for building the PRS in the Korean study were derived from the discovery data [26]. In this regard, a slightly improvement of the aROC was also obtained when we evaluated the w-PRS (0.57); nevertheless, our data was also used as discovery data by Huyghe et al. [7], and for this reason, the unweighted PRS was selected to conduct all the analyses.

The performance of the PRS was very similar in women (0.57) and men (0.56). This is in agreement with previous studies which have observed either better performances in men or no differences by sex [27]. Regarding age, the discriminatory accuracy of the model among participants ≥ 60 years was slightly better than younger participants < 60 years (0.54 and 0.57, respectively). Despite that the incidence of CRC is increasing in younger populations (< 50 years), the probability of having a lesion increases with age [28]. In Spain, the target population to become eligible to participate in CRC screening programs is the group aged 50–69. We have shown that higher PRS values are associated with higher relative risks of disease and that higher PPV values are observed for elderly participants; however, there were no differences in the PRS values by age (< 60 vs. ≥ 60). This result may indicate that the current selected age range to participate is not optimal. The scientific community is discussing whether people at 45 should begin CRC screening and discontinue it at the age of 75, as half of CRC cases are diagnosed around 70 [24, 29, 30]; however, we could not investigate these age groups as they do not meet our screening program inclusion criteria. Though the PRS has shown to be robust in the diverse subgroup analyses, only 26 SNPs were statistically significant (Additional file 1: Tab. S1). This probably was related to the limited sample size of our study compared to the GWAS studies in which the SNPs were discovered [7]. Most recently discovered SNPs have small MAF and small effect and, thus, they may show high variability in smaller sample sizes. Other factor that may explain the relatively small number of significant SNPs is the definition of the outcome. The SNPs used for the PRS have been discovered in studies of invasive CRC, but in our group of cases, we included subjects with non-invasive high-risk lesions which, as we have shown, have lower average PRS, and thus reduce the power to observe significant effects.

CRC screening in Spain is based on FIT test. Since the present study includes screening participants, it is not surprising that most of our participants within the screening subset had a positive test result (77%). We had a smaller sample of subjects with negative FIT (*n* = 362), which may be more representative of the general population, since only 6% of the participants test positive. To our knowledge, no study has evaluated the hypothetic impact of incorporating the PRS into a CRC screening setting that uses FIT to indicate colonoscopy (Fig. 4). The overall NPV among negative FIT, which represents the general population, was 0.84. The NPV at the 10–90th PRS percentiles were 0.90–0.84, respectively. The PPV among negative FIT participants, which corresponded to those cases that do not bleed but have a lesion, ranged from 0.17 at the 10th percentile to 0.19 at the 90th percentile. It can be inferred from the above results that lower values of PRS seem to improve the general NPV from the overall population and could decrease the number of false negative results. Besides, the overall PPV among FIT+ was almost 0.50. The PPV at the 10–90th PRS percentiles were 0.48–0.57, respectively. The readout here could be that performing a PRS after a FIT+ result does not avoid FIT+ to be referred for colonoscopy, which is one of the main goals for risk stratification. As the number of SNPs increases, the PRS extremes get wider; however, there are still some cases (IRL, HRL, or CRC) that have low PRS values. At present, with the number of SNPs that are known to be independently associated with CRC, the PRS is not able to safely stratify the general population since the improvement on PPV values is still scare.

The major strength of the present study is the inclusion of all CRC screening and non-screening scenarios: from population-based controls, non-advanced lesions, advanced lesions to clinically diagnosed CRC. In this sense, all participants recruited from the ongoing CRC Screening Program and included in the present study were referred for colonoscopy examination despite of their FIT result. This allowed us to classify participants following the proposal by Castells et al. [14]. Additionally, we have used the most recent set of known independent signals for CRC [6]. Notwithstanding its strengths, there are some limitations in our study that should be mentioned. We could not perform a validation analysis, and the predictive accuracy was only adjusted using cross-validation. The sample size in the negative FIT result group was relatively small, and few lesions were detected, because the FIT test has good accuracy. This has led to wide confidence intervals in the predictive values. Moreover, since we stratified the sampling of our population by FIT, we could not directly estimate FIT sensibility and specificity. We adjusted for family history, but participants with a history of familial CRC or one first degree relative younger than 60 would have been excluded from the CRC Screening Program and referred to the gastroenterologist. Thus, we do not have this population to study, but we have shown elsewhere that the PRS has a similar contribution in familial CRC [31]. Also, despite study participants were predominantly of European ancestry (97%), all the analyses were adjusted by five principal components obtained from the AIMS. In this regard, our results should be interpreted with caution when extrapolated to populations that are more diverse.

## Conclusions

In conclusion, our study provides evidence that genetic susceptibility, assessed with the PRS, plays an important role along the CRC tumorigenesis pathway; however, in practice, its utility to stratify the general population or as a second test after a positive FIT result is still doubtful with the current number of SNPs. Thus, further studies with larger sample sizes, larger number of informative SNPs, and possibly other biomarkers (i.e., metabolome, microbiome) are warranted to discern whether the PRS is worth implementing in the clinical practice.

## Supplementary Information

**Additional file 1: Tab. S1**. Detailed information of the 133 SNPs used to calculate the PRS.

**Additional file 2: Fig. S1** Ancestry PCA of HapMap and our samples. **Fig. S2** Distribution of the polygenic risk score according to the different 7 risk groups by sex. Fig. S3 Distribution of the polygenic risk score according to the different 7 risk groups by age. **Fig. S4** Distribution of the adjusted polygenic risk score and the weighted polygenic risk score according to the different 7 risk groups. **Fig. S5** Distribution of the adjusted polygenic risk score and distribution of the polygenic risk score excluding non-European samples according to the different 7 risk groups. **Fig. S6** Analysis of the weighted adjusted polygenic risk score. **Fig. S7** Analysis of high-risk lesions and CRC cases. **Fig. S8** Risk of premalignant polyps and colorectal cancer risk among screening population according to the adjusted polygenic risk score. **Fig. S9** Distribution of the adjusted polygenic risk score, Receiver Operating Characteristic curve, and positive and negative predicted values for the number of CRC risk alleles by sex. **Fig. S10** Distribution of the adjusted polygenic risk score, Receiver Operating Characteristic curve, and positive and negative predicted values for the number of CRC risk alleles by age.

**Additional file 3: Tab. S2**. Sample size for the distribution of the PRS in cases and controls for each analyzed model.

**Additional file 4: Tab. S3** Detailed positive and negative predicted values and their correspondent 95%CI for each analyzed model.

## Data Availability

The datasets generated and analyzed in our study are available on reasonable request from the corresponding author (VM2, email: v.moreno@iconcologia.net).

## Abbreviations

95%CI: 95% Confidence intervals
AIMS: Ancestry-informative marker SNPs
aROC: Area under the receiver operating characteristics curve
COLSCREEN: Colorectal cancer screening study
CRC: Colorectal cancer
CRCGEN: Colorectal cancer genetics & genomics study
FIT: Fecal immunochemical test
FOBT: Fecal occult blood test
GWASs: Genome-wide association studies
HRL: High-risk lesions
IRL: Intermediate-risk lesions
LRL: Low-risk lesions
NPV: Negative predictive values
OR: Odds ratios
PCA: Principal component analysis
PPV: Positive predictive values
PRS: Polygenic risk scores
SNPs: Single nucleotide polymorphisms
w-PRS: Weighted PRS

## References

[1] World Health Organization International Agency for Research on Cancer (IARC). Global Cancer Observatory. Cancer Today. 2020.

[2] Lansdorp-Vogelaar I, Knudsen AB, Brenner H (2010). Cost-effectiveness of colorectal cancer screening - an overview. Best Pract Res Clin Gastroenterol..

[3] IARC Working Group on the Evaluation of Cancer-Preventive Interventions. Colorectal Cancer Screening. 2019.

[4] Keum N, Giovannucci E (2019). Global burden of colorectal cancer: emerging trends, risk factors and prevention strategies. Nat Rev Gastroenterol Hepatol..

[5] Darbà J, Marsà A (2020). Results after 10 years of colorectal cancer screenings in Spain: hospital incidence and in-hospital mortality (2011-2016). PLoS One..

[6] Thomas M, Sakoda LC, Hoffmeister M, Rosenthal EA, Lee JK, van Duijnhoven FJB, Platz EA, Wu AH, Dampier CH, de la Chapelle A, Wolk A, Joshi AD, Burnett-Hartman A, Gsur A, Lindblom A, Castells A, Win AK, Namjou B, van Guelpen B, Tangen CM, He Q, Li CI, Schafmayer C, Joshu CE, Ulrich CM, Bishop DT, Buchanan DD, Schaid D, Drew DA, Muller DC, Duggan D, Crosslin DR, Albanes D, Giovannucci EL, Larson E, Qu F, Mentch F, Giles GG, Hakonarson H, Hampel H, Stanaway IB, Figueiredo JC, Huyghe JR, Minnier J, Chang-Claude J, Hampe J, Harley JB, Visvanathan K, Curtis KR, Offit K, Li L, le Marchand L, Vodickova L, Gunter MJ, Jenkins MA, Slattery ML, Lemire M, Woods MO, Song M, Murphy N, Lindor NM, Dikilitas O, Pharoah PDP, Campbell PT, Newcomb PA, Milne RL, MacInnis RJ, Castellví-Bel S, Ogino S, Berndt SI, Bézieau S, Thibodeau SN, Gallinger SJ, Zaidi SH, Harrison TA, Keku TO, Hudson TJ, Vymetalkova V, Moreno V, Martín V, Arndt V, Wei WQ, Chung W, Su YR, Hayes RB, White E, Vodicka P, Casey G, Gruber SB, Schoen RE, Chan AT, Potter JD, Brenner H, Jarvik GP, Corley DA, Peters U, Hsu L (2020). Genome-wide modeling of polygenic risk score in colorectal cancer risk. Am J Hum Genet..

[7] Huyghe JR, Bien SA, Harrison TA, Kang HM, Chen S, Schmit SL, Conti DV, Qu C, Jeon J, Edlund CK, Greenside P, Wainberg M, Schumacher FR, Smith JD, Levine DM, Nelson SC, Sinnott-Armstrong NA, Albanes D, Alonso MH, Anderson K, Arnau-Collell C, Arndt V, Bamia C, Banbury BL, Baron JA, Berndt SI, Bézieau S, Bishop DT, Boehm J, Boeing H, Brenner H, Brezina S, Buch S, Buchanan DD, Burnett-Hartman A, Butterbach K, Caan BJ, Campbell PT, Carlson CS, Castellví-Bel S, Chan AT, Chang-Claude J, Chanock SJ, Chirlaque MD, Cho SH, Connolly CM, Cross AJ, Cuk K, Curtis KR, de la Chapelle A, Doheny KF, Duggan D, Easton DF, Elias SG, Elliott F, English DR, Feskens EJM, Figueiredo JC, Fischer R, FitzGerald LM, Forman D, Gala M, Gallinger S, Gauderman WJ, Giles GG, Gillanders E, Gong J, Goodman PJ, Grady WM, Grove JS, Gsur A, Gunter MJ, Haile RW, Hampe J, Hampel H, Harlid S, Hayes RB, Hofer P, Hoffmeister M, Hopper JL, Hsu WL, Huang WY, Hudson TJ, Hunter DJ, Ibañez-Sanz G, Idos GE, Ingersoll R, Jackson RD, Jacobs EJ, Jenkins MA, Joshi AD, Joshu CE, Keku TO, Key TJ, Kim HR, Kobayashi E, Kolonel LN, Kooperberg C, Kühn T, Küry S, Kweon SS, Larsson SC, Laurie CA, le Marchand L, Leal SM, Lee SC, Lejbkowicz F, Lemire M, Li CI, Li L, Lieb W, Lin Y, Lindblom A, Lindor NM, Ling H, Louie TL, Männistö S, Markowitz SD, Martín V, Masala G, McNeil CE, Melas M, Milne RL, Moreno L, Murphy N, Myte R, Naccarati A, Newcomb PA, Offit K, Ogino S, Onland-Moret NC, Pardini B, Parfrey PS, Pearlman R, Perduca V, Pharoah PDP, Pinchev M, Platz EA, Prentice RL, Pugh E, Raskin L, Rennert G, Rennert HS, Riboli E, Rodríguez-Barranco M, Romm J, Sakoda LC, Schafmayer C, Schoen RE, Seminara D, Shah M, Shelford T, Shin MH, Shulman K, Sieri S, Slattery ML, Southey MC, Stadler ZK, Stegmaier C, Su YR, Tangen CM, Thibodeau SN, Thomas DC, Thomas SS, Toland AE, Trichopoulou A, Ulrich CM, van den Berg DJ, van Duijnhoven FJB, van Guelpen B, van Kranen H, Vijai J, Visvanathan K, Vodicka P, Vodickova L, Vymetalkova V, Weigl K, Weinstein SJ, White E, Win AK, Wolf CR, Wolk A, Woods MO, Wu AH, Zaidi SH, Zanke BW, Zhang Q, Zheng W, Scacheri PC, Potter JD, Bassik MC, Kundaje A, Casey G, Moreno V, Abecasis GR, Nickerson DA, Gruber SB, Hsu L, Peters U (2019). Discovery of common and rare genetic risk variants for colorectal cancer. Nat Genet..

[8] Saunders CL, Kilian B, Thompson DJ, McGeoch LJ, Griffin SJ, Antoniou AC (2020). External validation of risk prediction models incorporating common genetic variants for incident colorectal cancer using UK Biobank. Cancer Prev Res (Phila)..

[9] Balavarca Y, Weigl K, Thomsen H, Brenner H (2020). Performance of individual and joint risk stratification by an environmental risk score and a genetic risk score in a colorectal cancer screening setting. Int J Cancer..

[10] Erben V, Carr PR, Guo F, Weigl K, Hoffmeister M, Brenner H. Individual and joint associations of genetic risk and healthy lifestyle score with colorectal neoplasms among participants of screening colonoscopy. Cancer Prev Res (Phila). 2021;14:649–58. 10.1158/1940-6207.CAPR-20-0576 33653736

[11] Northcutt MJ, Shi Z, Zijlstra M, Shah A, Zheng S, Yen EF, Khan O, Beig MI, Imas P, Vanderloo A, Ansari O, Xu J, Goldstein JL (2021). Polygenic risk score is a predictor of adenomatous polyps at screening colonoscopy. BMC Gastroenterol..

[12] Peris M, Espinàs JA, Muñoz L, Navarro M, Binefa G, Borràs JM, Catalan Colorectal Cancer Screening Pilot Programme Group (2007). Lessons learnt from a population-based pilot programme for colorectal cancer screening in Catalonia (Spain). J Med Screen..

[13] Binefa G, Garcia M, Milà N, Fernández E, Rodríguez-Moranta F, Gonzalo N, Benito L, Clopés A, Guardiola J, Moreno V (2016). Colorectal cancer screening programme in Spain: results of key performance indicators after five rounds (2000-2012). Sci Rep..

[14] Castells A, Andreu M, Binefa G, Fité A, Font R, Espinàs JA (2015). Postpolypectomy surveillance in patients with adenomas and serrated lesions: a proposal for risk stratification in the context of organized colorectal cancer-screening programs. Endoscopy..

[15] Das S, Forer L, Schönherr S, Sidore C, Locke AE, Kwong A, Vrieze SI, Chew EY, Levy S, McGue M, Schlessinger D, Stambolian D, Loh PR, Iacono WG, Swaroop A, Scott LJ, Cucca F, Kronenberg F, Boehnke M, Abecasis GR, Fuchsberger C (2016). Next-generation genotype imputation service and methods. Nat Genet..

[16] Altshuler DM, Gibbs RA, Peltonen L, Altshuler DM, Gibbs RA, International HapMap 3 Consortium (2010). Integrating common and rare genetic variation in diverse human populations. Nature.

[17] Mann HB. Nonparametric tests against trend. Econometrica: Journal of the econometric society. JSTOR; 1945;245–59.

[18] Kendall MG. Rank correlation methods. Griffin; 1948;

[19] Robin X, Turck N, Hainard A, Tiberti N, Lisacek F, Sanchez J-C, Müller M (2011). pROC: an open-source package for R and S+ to analyze and compare ROC curves. BMC Bioinformatics..

[20] Chang CC, Chow CC, Tellier LC, Vattikuti S, Purcell SM, Lee JJ (2015). Second-generation PLINK: rising to the challenge of larger and richer datasets. Gigascience..

[21] Dunlop MG, Tenesa A, Farrington SM, Ballereau S, Brewster DH, Koessler T, Pharoah P, Schafmayer C, Hampe J, Völzke H, Chang-Claude J, Hoffmeister M, Brenner H, von Holst S, Picelli S, Lindblom A, Jenkins MA, Hopper JL, Casey G, Duggan D, Newcomb PA, Abulí A, Bessa X, Ruiz-Ponte C, Castellví-Bel S, Niittymäki I, Tuupanen S, Karhu A, Aaltonen L, Zanke B, Hudson T, Gallinger S, Barclay E, Martin L, Gorman M, Carvajal-Carmona L, Walther A, Kerr D, Lubbe S, Broderick P, Chandler I, Pittman A, Penegar S, Campbell H, Tomlinson I, Houlston RS (2013). Cumulative impact of common genetic variants and other risk factors on colorectal cancer risk in 42,103 individuals. Gut..

[22] Hsu L, Jeon J, Brenner H, Gruber SB, Schoen RE, Berndt SI (2015). A model to determine colorectal cancer risk using common genetic susceptibility loci. Gastroenterology.

[23] Ibáñez-Sanz G, Díez-Villanueva A, Alonso MH, Rodríguez-Moranta F, Pérez-Gómez B, Bustamante M, Martin V, Llorca J, Amiano P, Ardanaz E, Tardón A, Jiménez-Moleón JJ, Peiró R, Alguacil J, Navarro C, Guinó E, Binefa G, Fernández-Navarro P, Espinosa A, Dávila-Batista V, Molina AJ, Palazuelos C, Castaño-Vinyals G, Aragonés N, Kogevinas M, Pollán M, Moreno V (2017). Risk model for colorectal cancer in Spanish population using environmental and genetic factors: results from the MCC-Spain study. Sci Rep..

[24] Jeon J, Du M, Schoen RE, Hoffmeister M, Newcomb PA, Berndt SI (2018). Determining risk of colorectal cancer and starting age of screening based on lifestyle, environmental, and genetic factors. Gastroenterology.

[25] Jia G, Lu Y, Wen W, Long J, Liu Y, Tao R (2020). Evaluating the utility of polygenic risk scores in identifying high-risk individuals for eight common cancers. JNCI Cancer Spectr.

[26] Jo J, Nam CM, Sull JW, Yun JE, Kim SY, Lee SJ, Kim YN, Park EJ, Kimm H, Jee SH (2012). Prediction of colorectal cancer risk using a genetic risk score: the Korean Cancer Prevention Study-II (KCPS-II). Genomics Inform..

[27] Usher-Smith JA, Harshfield A, Saunders CL, Sharp SJ, Emery J, Walter FM, et al. External validation of risk prediction models for incident colorectal cancer using UK Biobank. Br J Cancer. 2018;118:750–9. 10.1038/bjc.2017.463 PMC5846069 29381683

[28] Araghi M, Soerjomataram I, Bardot A, Ferlay J, Cabasag CJ, Morrison DS, de P, Tervonen H, Walsh PM, Bucher O, Engholm G, Jackson C, McClure C, Woods RR, Saint-Jacques N, Morgan E, Ransom D, Thursfield V, Møller B, Leonfellner S, Guren MG, Bray F, Arnold M (2019). Changes in colorectal cancer incidence in seven high-income countries: a population-based study. Lancet Gastroenterol Hepatol..

[29] Mannucci A, Zuppardo RA, Rosati R, Leo MD, Perea J, Cavestro GM (2019). Colorectal cancer screening from 45 years of age: thesis, antithesis and synthesis. World J Gastroenterol..

[30] Qaseem A, Crandall CJ, Mustafa RA, Hicks LA, Wilt TJ (2019). Clinical Guidelines Committee of the American College of Physicians. Screening for colorectal cancer in asymptomatic average-risk adults: a guidance statement from the American College of Physicians. Ann Intern Med..

[31] Mur P, Bonifaci N, Díez-Villanueva A, Munté E, Alonso MH, Obón-Santacana M (2021). Non-lynch familial and early-onset colorectal cancer explained by accumulation of low-risk genetic variants. Cancers (Basel).

