# Supplementary material for: Polygenic risk score across distinct colorectal cancer screening outcomes: from premalignant polyps to colorectal cancer
Source: BMC Med. 2021 Nov 8;19:261. doi: 10.1186/s12916-021-02134-x (PMC8574048; doi:10.1186/s12916-021-02134-x)

Polygenic risk score across distinct colorectal cancer screening outcomes: from premalignant polyps to colorectal cancer.

Mireia Obón-Santacana [1,2,3], Anna Díez-Villanueva[1,2,3], Maria Henar Alonso[1,2,3], Gemma Ibáñez-Sanz[1,2,3,4], Elisabet Guinó[1,2,3], Ana López[1,2,3], Lorena Rodríguez-Alonso[4], Alfredo Mata[5], Ana García-Rodríguez[6], Andrés García Palomo[7], Antonio J Molina[8,9], Montse Garcia[3,10,11], Gemma Binefa[3,10,11], Vicente Martín[3,8,9], Victor Moreno[1,2,3,12,*]

__Supplementary Figure Captions__

Supplementary Figure 1 Ancestry PCA of HapMap and our samples

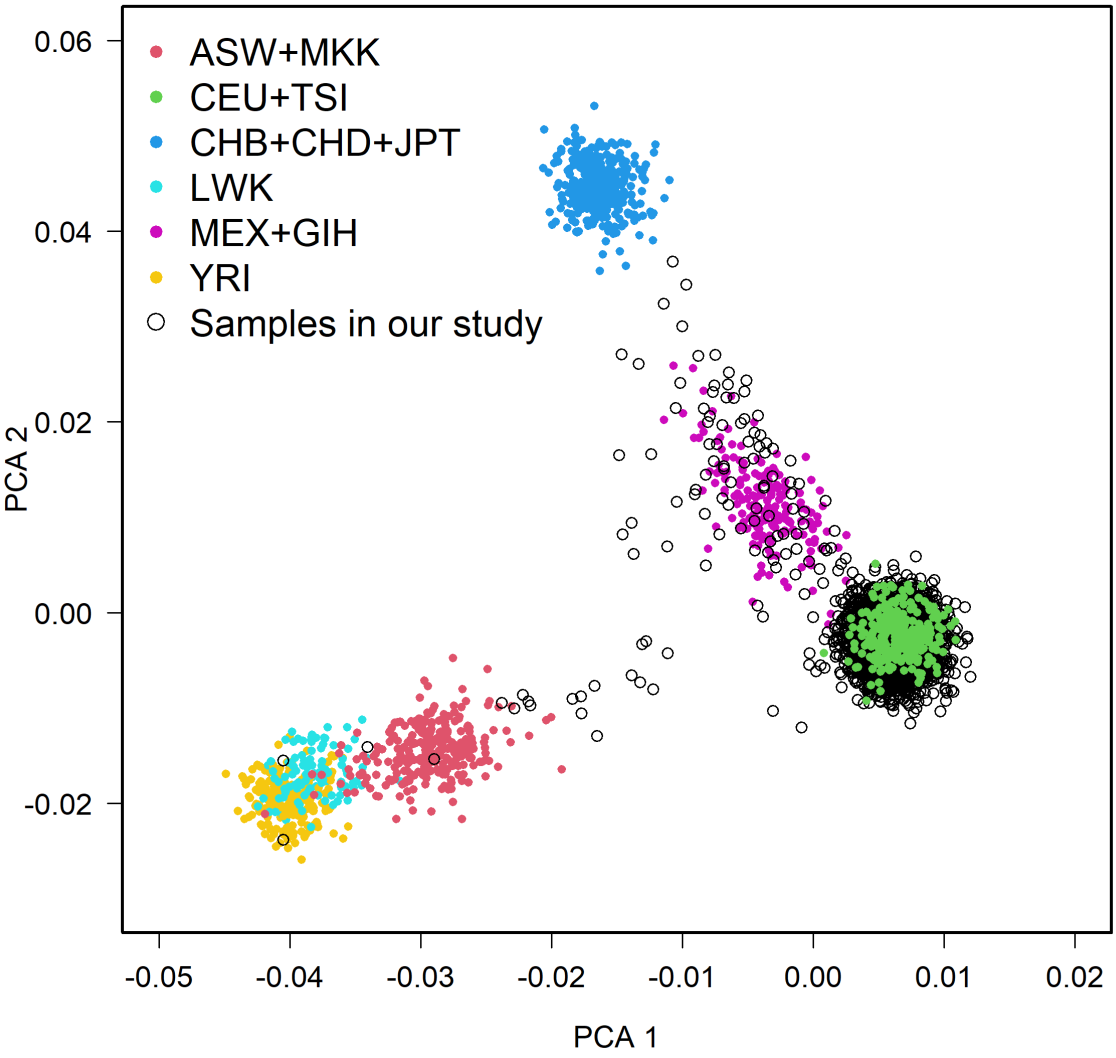

Supplementary Figure 1 legend: **ASW**: African ancestry in Southwest USA; **CEU**: Utah residents with Northern and Western European ancestry from the CEPH collection, **CHB**: Han Chinese in Beijing, China, **CHD:** Chinese in Metropolitan Denver, Colorado, **GIH:** Gujarati Indians in Houston, Texas, **JPT:** Japanese in Tokyo, Japan, **LWK:** Luhya in Webuye, Kenya, **MEX:** Mexican ancestry in Los Angeles, California, **MKK:** Maasai in Kinyawa, Kenya, **TSI:** Toscani in Italia and **YRI**: Yoruba in Ibadan, Nigeria.

Supplementary Figure 2 Distribution of the polygenic risk score according to the different 7 risk groups by sex.

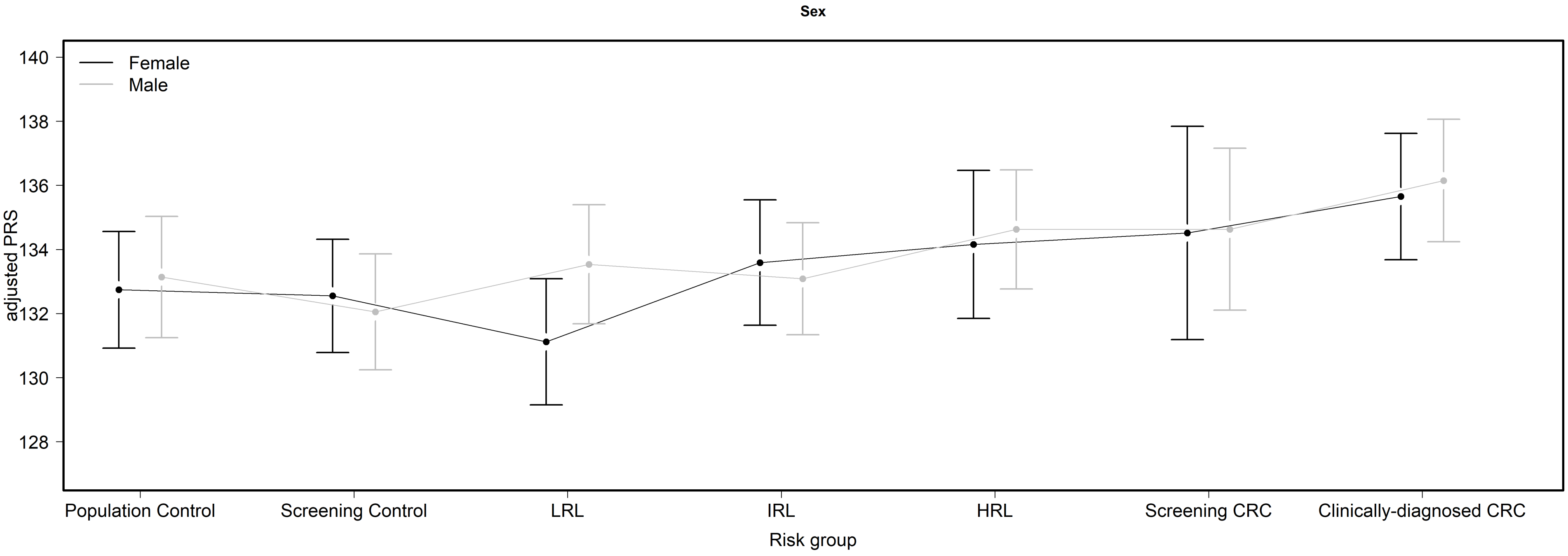

Supplementary Figure 2 legend: Data are represented as the mean and 95% CI for each group.

Supplementary Figure 3 Distribution of the polygenic risk score according to the different 7 risk groups by age

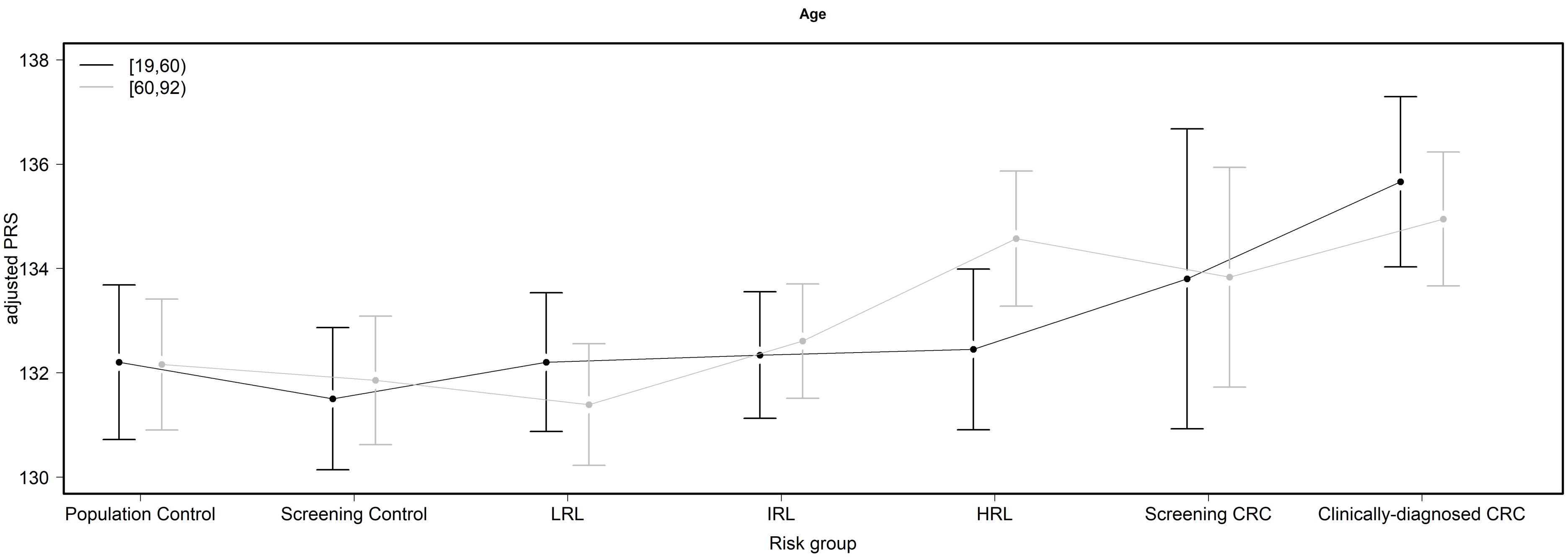

Supplementary Figure 3 legend: Data are represented as the mean and 95% CI for each group.

Supplementary Figure 4 Distribution of the adjusted polygenic risk score and the weighted polygenic risk score according to the different 7 risk groups.

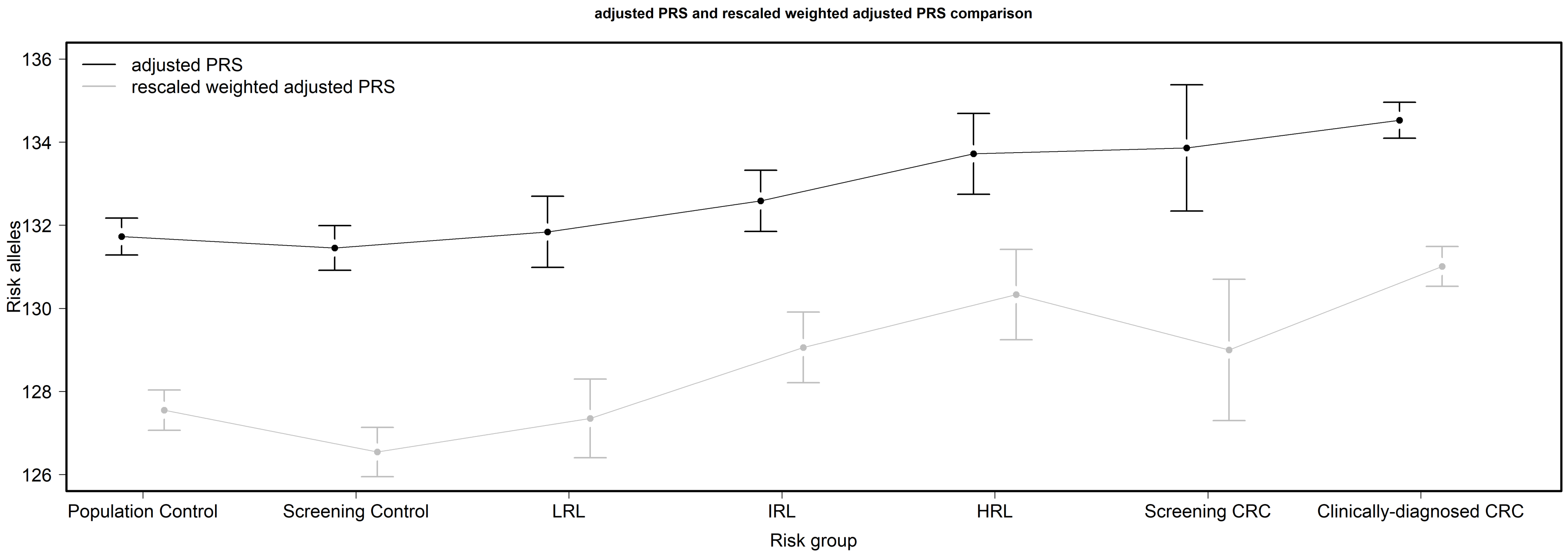

Supplementary Figure 4 legend: Data are represented as the mean and 95% CI for each risk group. To plot the weighted polygenic risk score in polygenic risk score scale, the weighted polygenic risk needed to be divided by the sum of the reported weights and multiplied by the total number of SNPs.

Supplementary Figure 5 Distribution of the adjusted polygenic risk score and distribution of the polygenic risk score excluding non-European samples according to the different 7 risk groups.

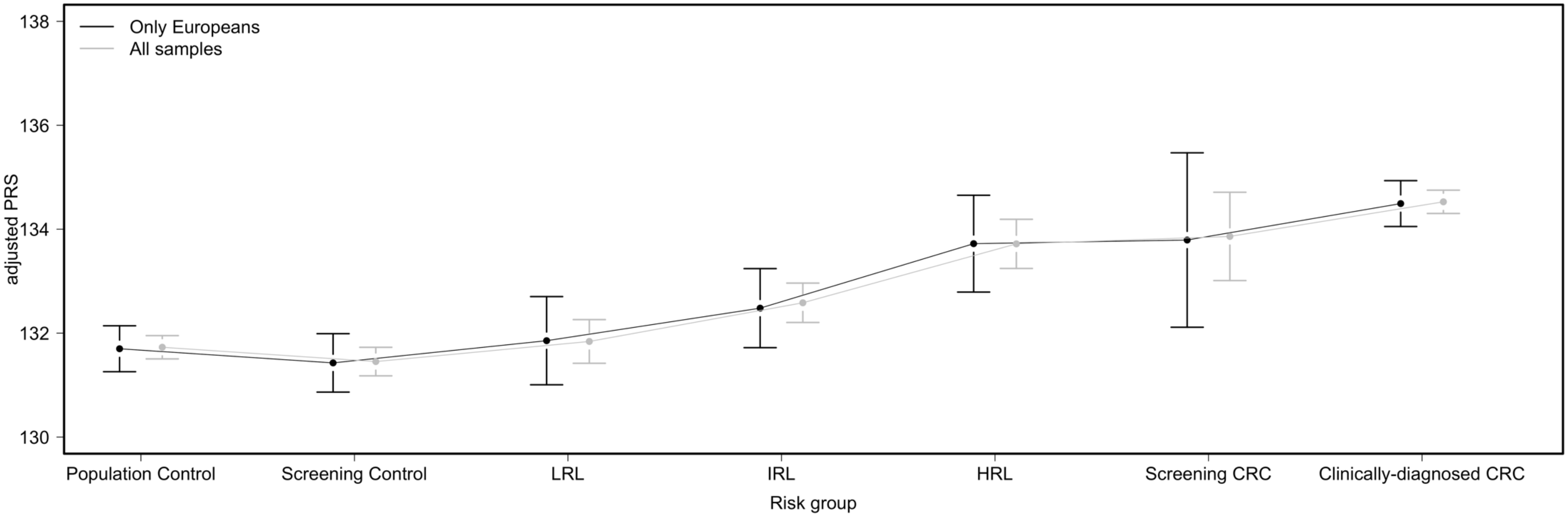

Supplementary Figure 5 legend: Data are represented as the mean and 95% CI for each group.

Supplementary Figure 6 Analysis of the weighted adjusted polygenic risk score (A) Distribution in cases and controls. (B) Receiver Operating Characteristic curve based on 133 SNPs used to measure the area under the curve in cases compared to controls. (C) Positive and negative predicted values for the number of CRC risk alleles weighted by fecal immunochemical test (positive weight = 0.06 and negative weight = 0.94).

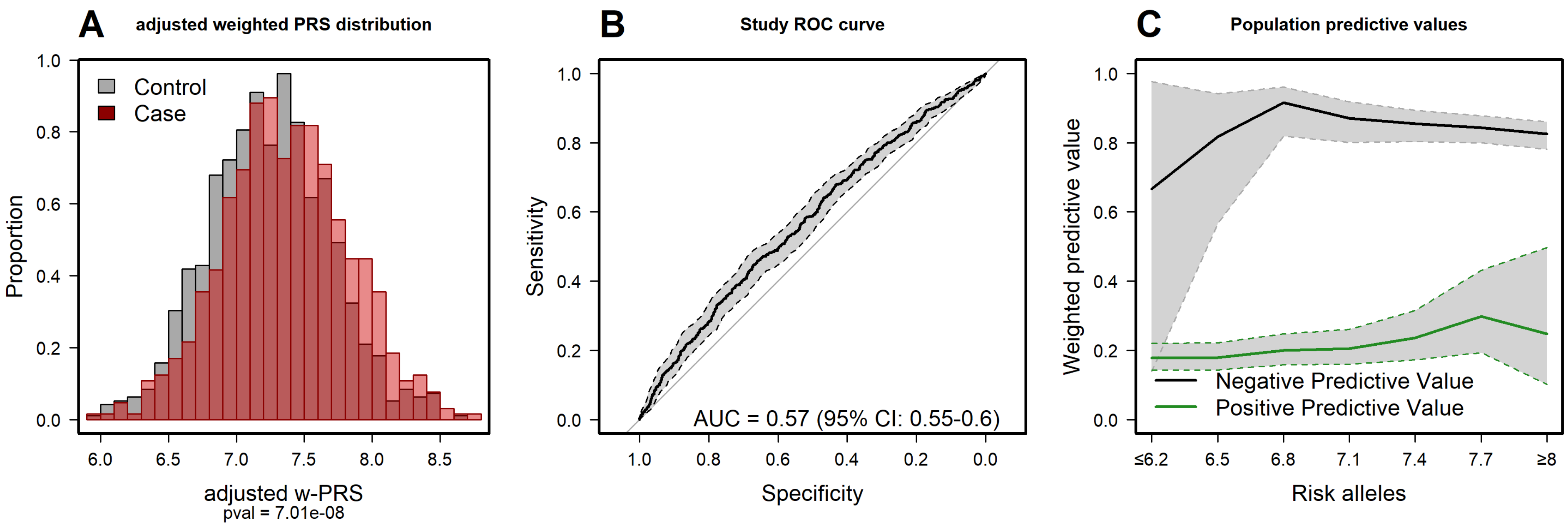

Supplementary Figure 7 Analysis of high-risk lesions and CRC cases (A) Distribution of the adjusted polygenic risk score in cases (high-risk lesions and CRC cases) and controls (low-risk lesions, intermediate-risk lesions, and controls). (B) Receiver Operating Characteristic curve based on 133 SNPs used to measure the area under the curve in cases compared to controls. (C) Positive and negative predicted values for the number of CRC risk alleles weighted by fecal immunochemical test (positive weight = 0.06 and negative weight = 0.94).

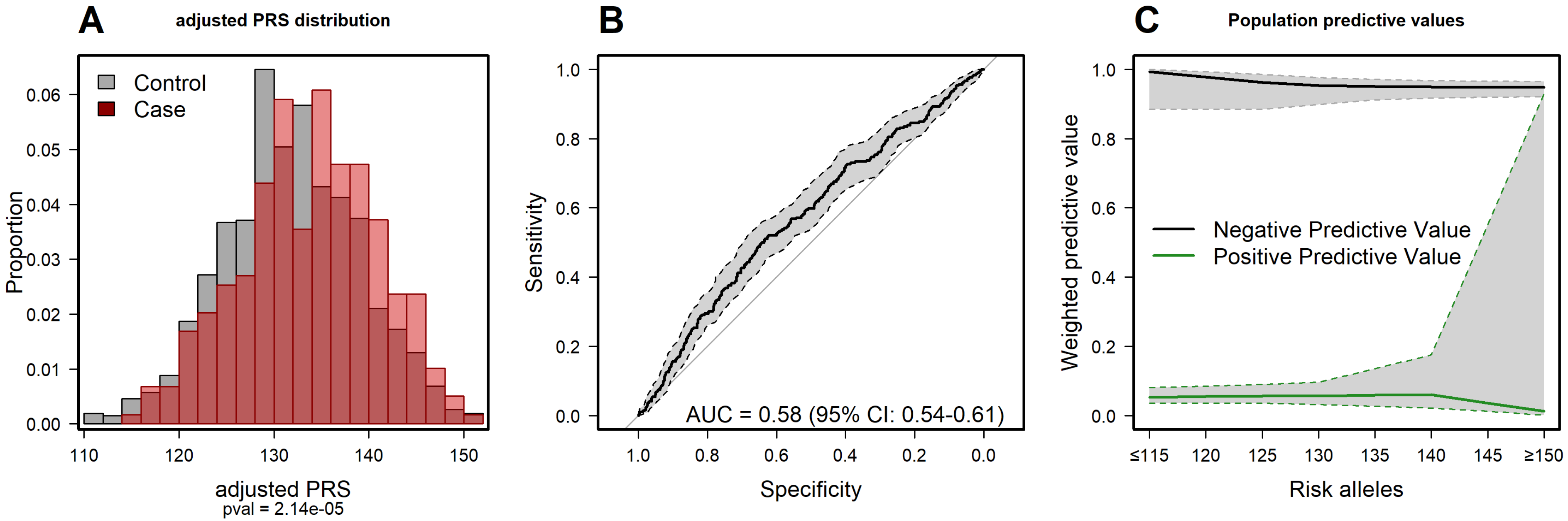

Supplementary Figure 8 Risk of premalignant polyps and colorectal cancer risk among screening population according to the adjusted polygenic risk score.

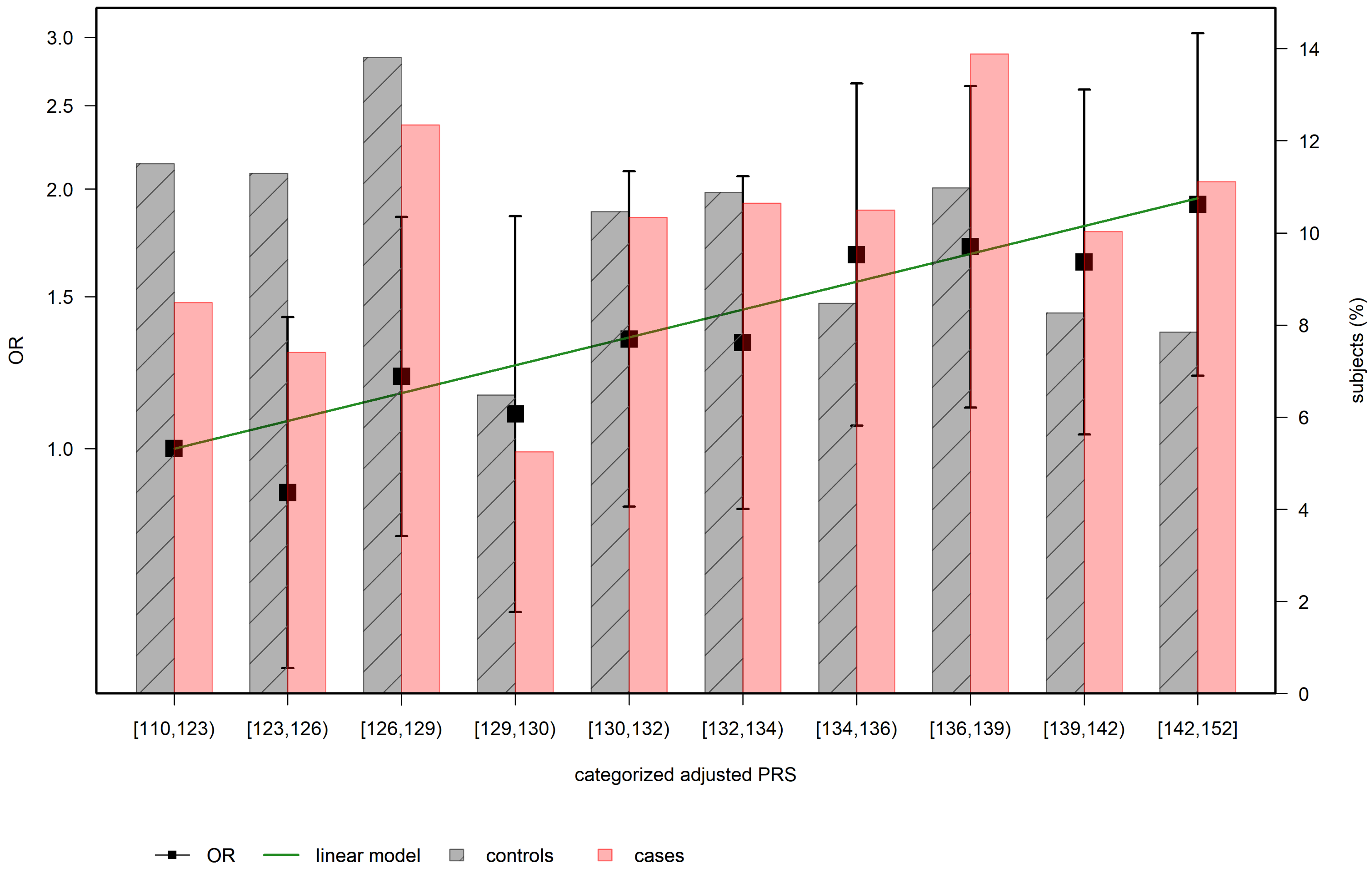

Supplementary Figure 9 (A) Distribution of the adjusted polygenic risk score among women in cases and controls. (B) Receiver Operating Characteristic curve for based on 133 SNPs used to measure the area under the curve in female cases compared to female controls. (C) Positive and negative predicted values for the number of CRC risk alleles in women weighted by fecal immunochemical test (positive weight = 0.06 and negative weight = 0.94). (D) Distribution of the polygenic risk score among men in cases and controls. (E) Receiver Operating Characteristic curve based on 133 SNPs used to measure the area under the curve in male cases compared to male controls (F) Positive and negative predicted values for the number of CRC risk alleles in men weighted by fecal immunochemical test (positive weight = 0.06 and negative weight = 0.94).

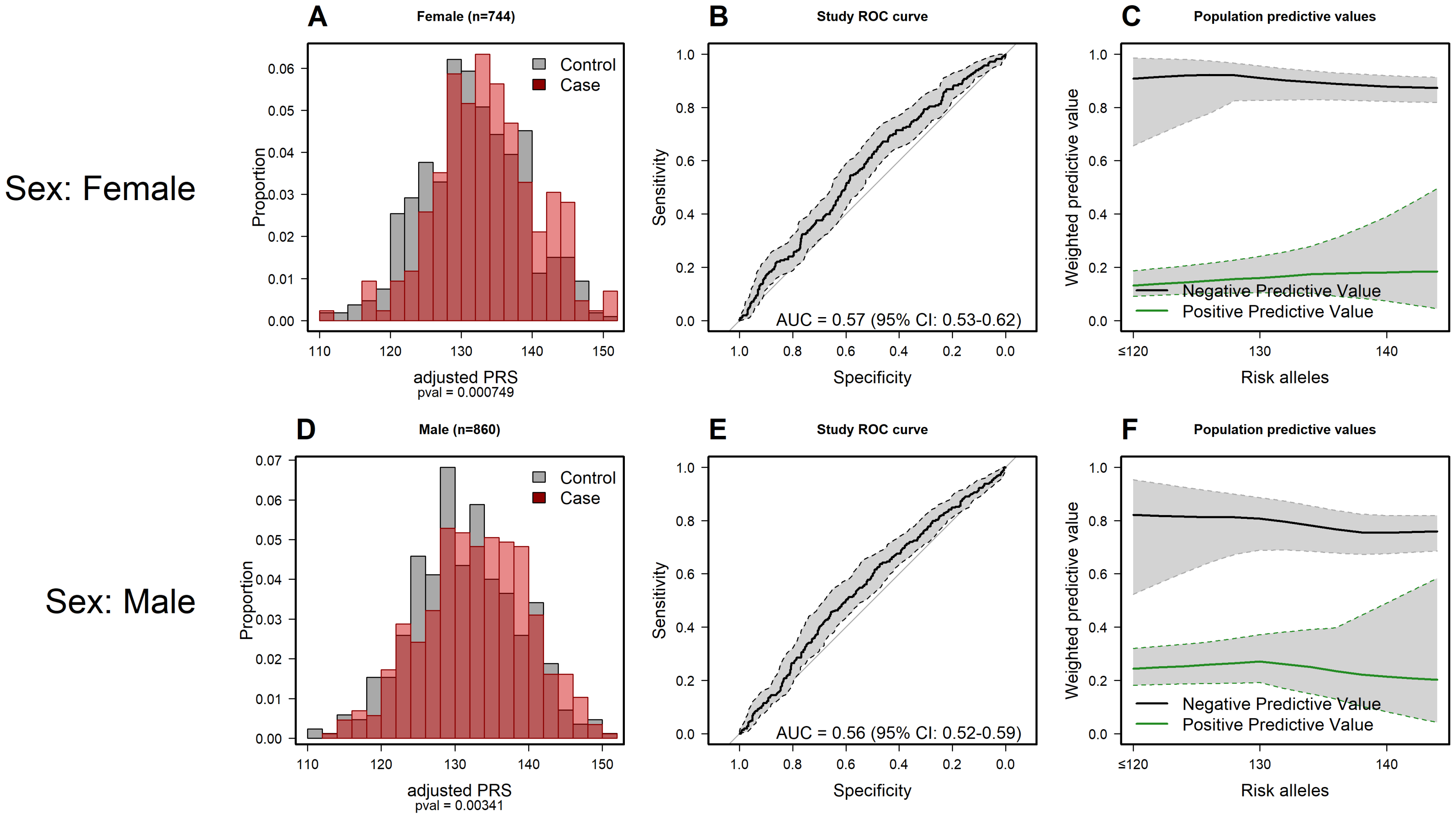

Supplementary Figure 10 (A) Distribution of the adjusted polygenic risk score among <60 years in cases and controls. (C) Receiver Operating Characteristic curve for based on 133 SNPs used to measure the area under the curve in <60 years cases compared to <60 years controls. (C) Positive and negative predicted values for the number of CRC risk alleles in <60y weighted by fecal immunochemical test (positive weight = 0.06 and negative weight = 0.94). (D) Distribution of the polygenic risk score among ≥60 years in cases and controls. (E) Receiver Operating Characteristic curve based on 133 SNPs used to measure the area under the curve in ≥60 years cases compared to ≥60 years controls (F) Positive and negative predicted values for the number of CRC risk alleles in ≥60y weighted by fecal immunochemical test (positive weight = 0.06 and negative weight = 0.94).

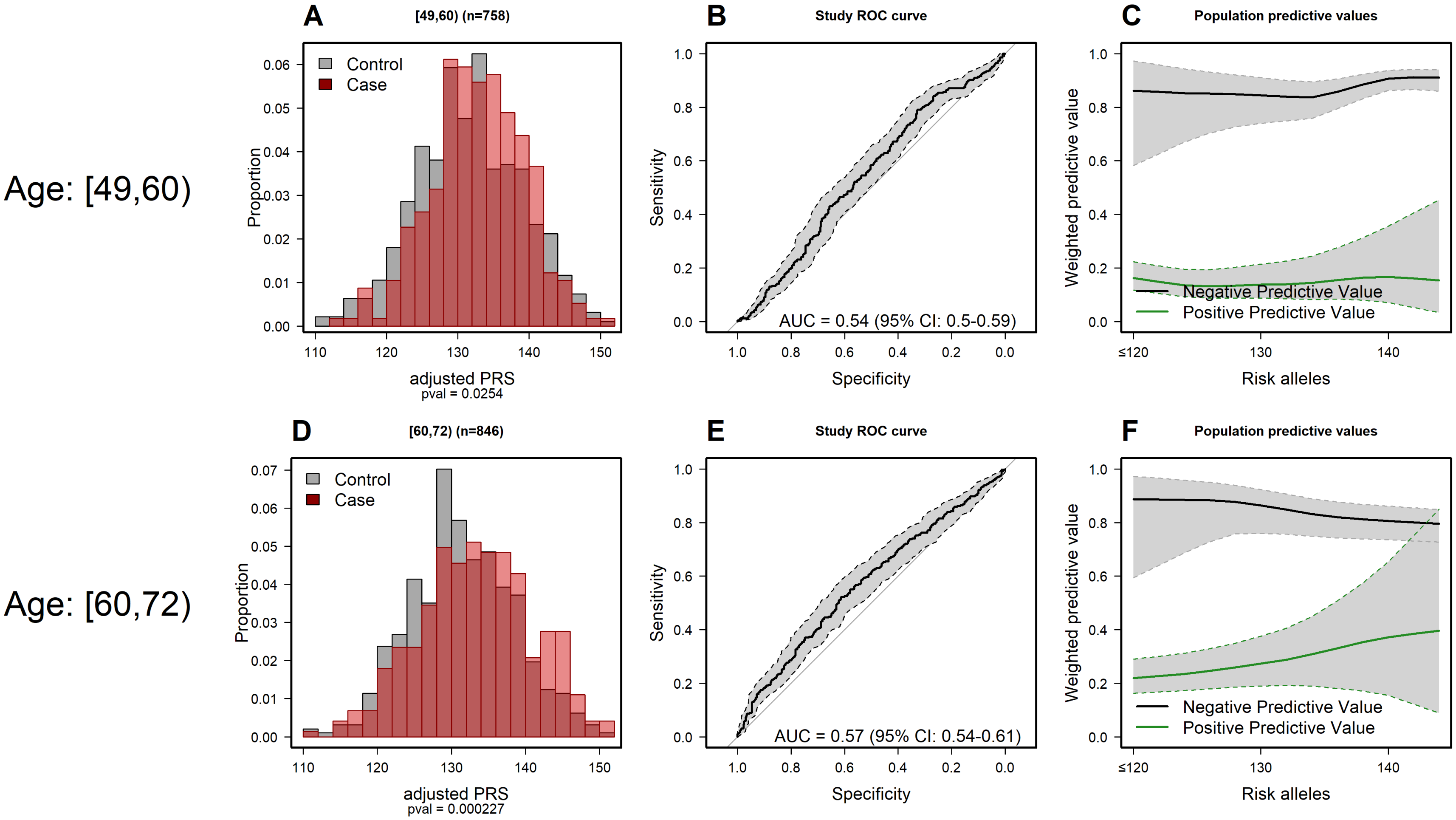

Supplement: Supplementary file 2 — Additional file 2: Fig. S1 Ancestry PCA of HapMap and our samples. Fig. S2 Distribution of the polygenic risk score according to the different 7 risk groups by sex. Fig. S3 Distribution of the polygenic risk score according to the different 7 risk groups by age. Fig. S4 Distribution of the adjusted polygenic risk score and the weighted polygenic risk score according to the different 7 risk groups. Fig. S5 Distribution of the adjusted polygenic risk score and distribution of the polygenic risk score excluding non-European samples according to the different 7 risk groups. Fig. S6 Analysis of the weighted adjusted polygenic risk score. Fig. S7 Analysis of high-risk lesions and CRC cases. Fig. S8 Risk of premalignant polyps and colorectal cancer risk among screening population according to the adjusted polygenic risk score. Fig. S9 Distribution of the adjusted polygenic risk score, Receiver Operating Characteristic curve, and positive and negative predicted values for the number of CRC risk alleles by sex. Fig. S10 Distribution of the adjusted polygenic risk score, Receiver Operating Characteristic curve, and positive and negative predicted values for the number of CRC risk alleles by age. [file 12916_2021_2134_MOESM2_ESM.pdf]
